# Is there an association between working conditions and health? An analysis of the Sixth European Working Conditions Survey data

**DOI:** 10.1371/journal.pone.0211294

**Published:** 2019-02-12

**Authors:** Nunzia Nappo

**Affiliations:** Department of Political Science, Università di Napoli “Federico II”, Napoli, Italia; University of Rome, ITALY

## Abstract

This paper analyses the association between working conditions and physical health using data from the Sixth European Working Conditions Survey (EWCS6) released in 2017. The econometric analysis uses two indicators to describe health status: self-assessed health (*SAH*), which is a subjective indicator of health; and an objective indicator of health (*SICK*), which is based on the occurrence of any illness or health problem that has lasted or is expected to last for more than 6 months. The theoretical hypotheses concerning the association between working conditions and *SAH* and the association between working conditions and *SICK* are tested using a standard ordered probit model and a standard probit model, respectively. The results show that encouraging working conditions, work environment, and job support are associated with both better self-assessed health and better objective health.

## Introduction

Given the continuous and rapid transformations in work and working conditions, among academics in the fields of epidemiology, psychology and sociology and policy makers, the debate on the potential effects of work on health has persisted. Recently, economists have also started studying the impacts of work and work-related factors on both physical and psychological health [1]. To what extent work may affect health significantly depends on working conditions, as “employment and working conditions have powerful effects on health and health equity” [2]. Following the ILO [3] definition, working conditions cover a “broad range of topics and issues, from working time (hours of work, rest periods, and work schedules) to remuneration, as well as the physical conditions and mental demands that exist in the workplace”. A previous literature [4] advises that adverse working conditions hurt health and, conversely, being employed with proper working conditions plays a protecting role for both physical and mental health.

This study focuses on some characteristics of work and working conditions that affect workers’ health. While many previous studies investigate only specific diagnoses, the main aim of this paper is studying the association between working conditions and general physical health among the EU28 using data from the Sixth European Working Conditions Survey (EWCS6) fielded in 2015 and released in 2017. The EWCS6 provides detailed information on a wide range of issues, including exposure to physical and psychosocial risks, work organization, work-life balance, and several measures of health. The econometric analysis uses two indicators to describe health: self-assessed health, which is a subjective indicator of health, and a more objective indicator of health based on the occurrence of any illness or health problem that has lasted or is expected to last for more than 6 months.

Empirical evidence on the effects of working conditions on health comes from two main models, the “demand-control-support” model and the “effort-reward imbalance model”. Those models, which were developed by Karasek [5] and Karasek and Theorell [6] and by Siegrist [7], study the impacts of working conditions on individual health. The “demand-control-support” model is based on the following key concepts: job demand, job decision latitude or job control and social support at work. Job demand can be physical (concerning manual work) and/or psychological (regarding the pace, quantity and difficulty of work). In line with Marchand et al. [8], job demand can be contractual too, and it refers to hours of working and irregular work timetable. Workers’ ability to schedule their own duties and to manage their skills constitutes “job decision latitude”. Social support can positively affect health, since positive relationships with colleagues and with superiors may compensate for demanding conditions. Adverse effects on health do not come from a particular characteristic of work, but rather from the balance between all demands related to work and workers’ abilities to deal with those demands. Karasek [5] and Karasek and Theorell [6] state that low control combined with high demands creates health risks. The authors hypothesize that the intrinsic effects of the work organization on health depend on individuals’ own characteristics. They show a high incidence of symptoms of heart disease among workers who report both low control and high demand.

Siegrist [7] underlines the importance of rewards rather than the control structure of work, and his model includes personal characteristics too. According to Siegrist [7], there are three potential channels for rewarding workers: 1) an adequate salary, 2) respect and support, and 3) job security and career opportunities. Negative effects on workers’ health are expected when there is an imbalance between the demands on them and the monetary and non-monetary rewards they receive. When workers experience high effort/low reward conditions, in the long term, they are exposed to disorders, such as cardiovascular disease and mental or physical health problems.

Numerous analyses tested the “demand-control-support” model and the “effort-reward imbalance” model and provided evidence in their favour. Bosma et al. [9] investigated the association between two alternative job stress models, the effort-reward imbalance model and the job strain model, and the risk of coronary heart disease among male and female British civil servants. The imbalance between personal efforts and rewards was associated with a 2.15-fold higher risk of new instances of coronary heart disease. Job strain and high job demands were not correlated with coronary heart disease. However, low job control was related with new disease instances. Cheng et al. [10] employed a sample of 21,290 American females and found that, examined separately, low job control, high job demands, and low work-related social support were associated with poor health at the baseline and greater functional declines over the four year follow-up period. When examined in combination, women with low job control, high job demands, and low work-related social support had the greatest functional declines. Ostry et al. [11] compare the predictive validity of the demand/control and effort reward/imbalance models for the self-reported health status and the self-reported presence of any chronic condition in a sample of former and current sawmill manufacturing workers. Their results show that the demand/control and effort/reward imbalance models separately predicted self-reported poor health statuses. The effort-reward imbalance model predicted the presence of chronic diseases, and the demand/control model did not. Niedhammer and Chea [12] find that psychosocial factors at work contribute to health, particularly to cardiovascular health. They perform cross sectional and prospective analyses using samples of 11,447 and 7,664 French workers, respectively. With respect to the cross-sectional analysis, for both men or women, the results show significant associations between psychological demands, decision latitude, social support, and physical demands and self-reported health. Meanwhile, the prospective analysis indicated that high psychological demands for both men and women and low decision authority for men predicted self-reported poor health. The same result occurred for women with respect to low social support and high physical demands. Warren et al. [13] focus on physical and psychosocial job characteristics as mediators in the relationship between socioeconomic status (SES) and health. They found that people with more physically and psychosocially demanding jobs have less favourable health outcomes. Datta Gupta and Kristensen [14] study whether a satisfactory work environment can promote employees’ health, even after controlling for their socioeconomic status and lifestyle factors. They employ samples of workers from Denmark, France and Spain. The results for all three countries show that a good perceived work environment is a significant determinant of workers’ health, even after controlling for unobserved heterogeneity and minimizing reverse causality. Both the “demand-control-support” model and the “effort-reward imbalance” model assume that the probability of health deterioration increases when imbalances are associated with deficient support at work and/or a feeling of job insecurity. With respect to the lack of support on the job, Väänänen et al. [15] focus on the following: 1) the subjective health effects of an organizational merger among employees who had experienced a change in their own job position, and 2) the effects of pre-merger social support at work on those who experienced changes in job positions and on their subjective health. The authors conclude that social support had a significant effect on the effects of the change in one’s job position. A decline in job position strongly increased the risk of poor subjective health after the merger. Weak organizational support was associated with impaired subjective health. With respect to job insecurity, the literature [16–17] shows that it deteriorates health, since it is a source of stress. Caroli and Godard [18] investigate the causal effect of perceived job insecurity on health. They use data from the EWCS (2010) and run a causal assessment of the impacts of perceived job insecurity on health. The authors conclude that when the potential endogeneity of job insecurity is not considered, job insecurity seems to deteriorate health. When job insecurity is accounted for, the results change. Then, job insecurity is confirmed to have a health deteriorating effect only for the probability of suffering from headaches or eyestrain and skin problems.

The original contribution of this paper comes from the use of the EWCS6 data to analyse the association between working conditions and health. To the best of our knowledge, it is the first time that this data have been employed for this kind of investigation. The paper enables the achievement of a broader picture of the relationship between working conditions and health among the EU28. This large sample represents a strength and a limitation of the paper. It is a strength because it provides a wide picture of the relationship between working conditions and health in the EU. For this reason, and since the sample is very large, the results could be considered general and valid for the EU28. Conversely, the large sample could be a limitation, since it aggregates different countries with different work-related features within working contexts that are sometimes dissimilar among them. The major limit of the paper is the assessment of the association between working conditions and physical health in the EU without establishing the direction of the causal link between the two.

The rest of the paper is organized as follows. The materials and methods section provides information on the data employed in the econometric analyses, and it describes the models and the dependent and independent variables. The results and discussion section provides the results and discusses them. Some concluding observations follow.

## Materials and methods

The Sixth European Working Conditions Survey provides the individual data that are employed in the econometric analysis. The data were accessed and downloaded via the UK Data Service (data set name 10.5255/UKDA-SN-8098-4). The terms of service for the website from which data was collected were complied. The sixth release of the Survey was fielded in 2015. Eurofound [19] provides an exhaustive description of the survey design. The Survey provides a detailed picture of Europe at work over time and across countries, occupations, genders and age groups and provides an overview of the working conditions in European countries. Approximately 43.000 workers aged 15 or over that were randomly selected were interviewed face-to-face. The questionnaire contains issues related to employment status, working time duration and organization, work organization, learning and training, physical and psychosocial risk factors, health and safety, work-life balance, worker participation, earnings and financial security, and health.

The sample includes 35 countries, including the EU28, Norway, Switzerland, Albania, the former Yugoslav Republic of Macedonia, Montenegro, Serbia and Turkey. A panel dimension is not available.

The econometric analysis focuses on the EU28: Austria, Belgium, Bulgaria, Czech Republic, Denmark, Germany, Estonia, Ireland, Greece, Spain, France, Croatia, Italy, Cyprus, Latvia, Lithuania, Luxembourg, Hungary, Malta, the Netherlands, Poland, Portugal, Romania, Slovenia, Slovakia, Finland, Sweden, and the United Kingdom. The sample includes both employed and self-employed workers; however, the econometric analysis focuses only on employed workers. After removing the unselected respondents and those with missing data for the dependent and independent variables, the final data set is a cross-section sample, and it consists of 18,958 observations for *SAH* estimates and 18,895 for *SICK* estimates. Tables A, B, C and D in S1 File provide the descriptive statistics for the sample.

### Dependent variables

The econometric analysis uses two dependent variables: 1) self-assessed health (*SAH*), and 2) the occurrence of any illness or health problem that has lasted or is expected to last for more than 6 months (*SICK*). *SAH* is a subjective indicator of health that was collected through individual interviews. Interviewees responded to the following question: “How is your health in general? Would you say it is …?”

Responses were expressed on a scale of values from one (very good) to five (very bad) and were grouped by aggregating (1) answers that express the first two values (very good and good health), (2) answers that express the value in the middle (fair health), and (3) answers that express the last two values (bad and very bad health). The *SAH* values were aggregated, since the very low percentages of the last two values—bad and very bad health—equal 2.39% and 0.28%, respectively, and it is necessary to have a clear distinction among broadly good, fair and broadly bad perceived health.

Self-assessed health is largely used in the literature as an appropriate aggregate of all aspects of health [20] and earlier studies have revealed *SAH* to be correlated with objective health measures such as mortality [21]. However, the probability of asserting good or bad health may be affected by individual reporting heterogeneity [22]. For this reason, the econometric analysis also includes *SICK*, which, compared to *SAH*, can be considered a more objective indicator of health. Interviewed responded to the following question: “Do you have any illness or health problem that has lasted or is expected to last for more than 6 months?”

Responses were expressed as “Yes” or “No”.

### Independent variables

The choice of proper explanatory variables has been oriented by the theory and by the aim of the paper. Due to the unavailability of data, most of the explanatory variables are only proxies for the theoretical categories described in the previous section. However, an attempt was made to choose regressors that would allow us to compare our results with previous studies’ results (see the Discussion section). The paper uses the *Demand-Control*-*Support* model [6] and the *Effort-Reward Imbalance* model [7] as theoretical references. Those models imply three main dimensions—demand, control and reward—including the concept of support at work and the sensation of job security.

With respect to demanding job conditions (job pressure), we consider three covariates. 1) *Howmanyh* is a continuous variable that represents the number of hours the interviewee usually works per week in his/her main paid job. 2) *Notimef* is a dummy variable that equals 1 if the interviewee, in the last 12 months, found that her/his job prevented her/him from giving the time she/he wanted to her/his family, and it is 0 otherwise. 3) *Highspeed* represents whether the interviewee’s job involves working at a very high speed [18]. Seven answers were possible in a range from “all of the time” to “never”. The responses were combined in a summary scale that has been normalised to [0;10]. *Stress*, *Worrying* and *Exhausted* can be considered proxies for the psychological environment. *Stress* represents whether the worker experiences stress in his/her work. The responses, which were expressed using a five-point scale, were grouped by aggregating (1) “always” and “most of the time”, (2) “sometimes” and “rarely”, and (3) “never”. *Worrying* is a dummy equal to 1 if the interviewee, in the last 12 months, kept worrying (always, most of the time, or sometimes) about work when he/she was not working, and it is 0 otherwise (rarely or never). *Exhausted* reflects if workers felt exhausted at the end of the working day. The responses, which were expressed using a five-point scale, were grouped by aggregating (1) “always” and “most of the time”, (2) “sometimes and rarely”, and (3) “never”. As with previous studies, such as [13–14], we considered work satisfaction. *Satisfied* is a dummy variable that equals 1 if the interviewee is very satisfied or satisfied with the working conditions of his/her main paid job, and it is 0 otherwise (not very satisfied and not at all satisfied). We also considered *Inforisk* and *Hrisk*. The former regards the information on the health and safety risks related to the performance of his/her job that are available to the interviewee. *Inforisk* is a dummy that equals 1 if the interviewee is informed (very well informed or well informed), and it is 0 otherwise (not very well informed or not at all informed). *Hrisk* is a dummy variable that equals 1 if the interviewee thinks that his/her health or safety is at risk because of his/her work, and it is 0 otherwise.

*Envirconds* and *Physconds* are two covariates that reflect the harmful working conditions related to the work environment (exposure and involvement with adverse conditions) [18], which could imply risks from working. *Envirconds* is the aggregation of the following components: 1) vibrations from hand tools, machinery, etc.; 2) noise so loud that you would have to raise your voice to talk to people; 3) high temperatures that make you perspire, even when not working; 4) low temperatures, whether indoors or outdoors; 5) breathing in smoke, fumes (such as welding or exhaust fumes), powder, dust (such as wood dust or mineral dust), etc.; 6) breathing in vapours such as solvents and thinners; 7) handling or being in skin contact with chemical products or substances; 8) tobacco smoke from other people; and 9) handling or being in direct contact with materials that can be infectious, such as waste, bodily fluids, laboratory materials, etc. *Physconds* is the aggregation of the following components: 1) tiring or painful positions; 2) lifting or moving people; 3) carrying or moving heavy loads; 4) sitting; 5) repetitive hand or arm movements; 6) dealing directly with people who are not employees at your workplace such as customers, passengers, pupils, patients, etc.; 7) handling angry clients, customers, patients, pupils, etc.; 8) being in situations that are emotionally disturbing for you; and 9) working with computers, laptops, smartphones, etc. Both *Envirconds* and *Physconds* take values from 1 (all of the time) to 7 (never).

Other covariates represent encouragement, support that workers can enjoy on the job [5] and rewards [7]. Additionally, for those regressors, we use only proxies of the theoretical variables of the model. *Manhelp* specifies whether the manager helps and supports the interviewed workers. The answers, which were expressed using a range from “always” to “never”, were grouped by aggregating (1) “always” and “most of the time”, (2) “sometimes” and “rarely”, and (3) “never”. *Adcareer* reflects if the interviewee’s job offers good prospects for career advancement [18]. The responses, which were expressed using a five-point scale from “strongly agree” to “strongly disagree”, were grouped by aggregating (1) “strongly agree” and “tend to agree”, (2) “neither agree nor disagree”, and (3) “tend to disagree” and “strongly disagree”. *Recognition* specifies whether the worker receives the recognition that he/she deserves for his/her work. The responses, which were expressed using a five-point scale from “strongly agree” to “strongly disagree”, were grouped by aggregating (1) “strongly agree” and “tend to agree”, (2) “neither agree nor disagree”, and (3) “tend to disagree” and “strongly disagree”.

We controlled for some job characteristics too, including the following: 1) the sector within which workers perform their job, and 2) the type of occupation [18]. With respect to the sector, considering the public sector as the reference group, the regression includes two dummy variables: *private* equals 1 if the interviewee works in the private sector and 0 otherwise; and *other* equals 1 if the interviewed works in a joint private-public organization or company, the not-for-profit sector, an NGO or other, and it is 0 otherwise.

With respect to occupations, considering elementary occupations as the reference group, the regression includes the following dummies: 1) *armedforces* equals 1 if the worker is an armed forces occupation and 0 otherwise; 2) *managers* equals 1 if the worker is a manager and 0 otherwise; 3) *professionals* equals 1 if the worker is a professional and 0 otherwise; 4) *technicians* equals 1 if the worker is a technician and 0 otherwise; 5) *clerical* equals 1 if the worker is a clerical support worker and 0 otherwise; 6) *servicesales* equals 1 if the worker is a service and sales worker and 0 otherwise; 7) s*killedagriculturalforestryfish* equals 1 if the worker is a skilled agricultural, forestry or fishing worker and 0 otherwise; 8) *craftrades* equals 1 if the worker is a craft and related trades worker and 0 otherwise; 9) *plantmachine* equals 1 if the worker is a plant or machine operator or assemblers and 0 otherwise.

To avoid biased findings, we included country fixed effects in the empirical analysis. By considering the UK as the reference group, we included 27 country dummies in the regression.

Some standard socioeconomic control variables are included too. *Age* is a continuous variable. *Male* is a dummy that equals 1 if the interviewee is a male and 0 otherwise. *Phd* is a dummy variable that equals 1 if the interviewee has a doctoral degree and/or a Phd and 0 otherwise. The number of individuals living in the household is included as a continuous variable (*Npeople*). *Endmeet*, which is a proxy of income, is a dummy that equals 1 if the interviewee’s household total monthly income is able to make ends meet and 0 otherwise. *Endmeet* is preferred to income, since this income has numerous missing observations [18]. Table 1 provides a description of the covariates used in the empirical models; however, for brevity, it does not contain the 28 country dummies included in both models.

**Table 1:** Definition of the Independent Variables.

*Variable*	*Description—Question in the Survery*	
*Demographic*		
Age	Age in years at the time of the survey interview	Q2.b
Male	1 if male; 0 otherwise	Q2.a
Phd	1 if the interviewee has a degree and/or a Phd; 0 otherwise	Q106
Npeople	N. of people living in the household	Q1
Endmeet	1 if the interviewee household total monthly income is able to make ends meet; 0 otherwise	Q100
*Job demand*		
Howmanyh	N. of hours the interviewee usually works per week	Q24
Notimef	1 if the interviewee in the last 12 months found that her/his job prevented her/him from giving the time she/he wanted to her/his family, 0 otherwise	Q45c
Highspeed	Working at very high speed, 7-point scale [all of the time (1) to almost never (7)]	Q49a
*Psychological environment*		
Stress1	1 if the interviewee experiences stress in her/his work “always” and “most of the time”; 0 otherwise	Q61m
Stress2	1 if the interviewee “sometimes” and “rarely” experiences stress in her/his work; 0 otherwise	Q61m
Stress3	1 if the interviewee “never” experiences stress in her/his work; 0 otherwise	Q61m
Worrying	1 if the interviewee, in the last 12 months, kept worrying about work when he/she was not working; 0 otherwise	Q45a
Exhausted1	1 if the interviewee “always” and “most of the time” feels exhausted at the end of the working day; 0 otherwise	Q90d
Exhausted2	1 if the interviewee “sometimes” and “rarely” feels exhausted at the end of the working day; 0 otherwise	Q90d
Exhausted3	“1 if the interviewee “never” feels exhausted at the end of the working day; 0 otherwise	Q90d
Satisfied	1 if the interviewee is very satisfied and satisfied with working conditions; 0 otherwise	Q88
Inforisk	1 if the interviewee is informed on the health and safety risks related to the performance of his/her job; 0 otherwise	Q33
Hrisk	1 if the interviewee thinks her/his health or safety is at risk because of her/his work, 0 otherwise	Q73
*Job hazard*		
Envirconds	Working environmental conditions, 7-point scale [all of the time (1) to never (7)]	Q29
Physconds	Working physical conditions, 7-point scale [all of the time (1) to never (7)]	Q30
*Job recognition*		
Manhelp1	1 if the interviewee receives helps and supports by the manager “always” and “most of the time”; 0 otherwise	Q61b
Manhelp2	1 if the interviewee receives helps and supports by the manager “sometimes” and “rarely”; 0 otherwise	Q61b
Manhelp3	1 if the interviewee receives helps and supports by the manager “never”; 0 otherwise	Q61b
Adcareer1	1 if the interviewee “strongly agree” and “tend to agree” with the statement that her/his job offers good prospects for career advancement; 0 otherwise	Q89b
Adcareer2	1 if the interviewee “neither agrees nor disagrees” with the statement that her/his job offers good prospects for career advancement; 0 otherwise	Q89b
Adcareer3	1 if the interviewee “tend to disagree” and “strongly disagree” with the statement that her/his job offers good prospects for career advancement; 0 otherwise	Q89b
Recognition1	1 if the interviewee “strongly agree” and “tend to agree” with the statement that she/he receives the recognition she/he deserve for her/his work; 0 otherwise	Q89c
Recognition2	1 if the interviewee “neither agrees nor disagrees” with the statement that she/he receives the recognition he/she deserve for her/his work; 0 otherwise	Q89c
Recognition3	1 if the interviewee “tend to disagree” and “strongly disagree” with the statement that she/he receives the recognition he/she deserve for her/his work; 0 otherwise	Q89c
*Job characteristics*		
Private	1 if the interviewee works in the private sector; 0 otherwise	Q14
Public	1 if the interviewee works in the public sector; 0 otherwise	Q14
Other	1 if the interviewee works in a joint private-public organisation or company or the not-for-profit sector or an NGO or other; 0 otherwise	Q14
Armedforces	1 if the worker perform an armed forces occupation; 0 otherwise	Q5
Managers	1 if the worker is a manager; 0 otherwise	Q5
Professionals	1 if the worker is a professional; 0 otherwise	Q5
Technicians	1 if the worker is a technician; 0 otherwise	Q5
Clerical	1 if the worker is a clerical support worker; 0 otherwise	Q5
Servicesales	1 if the worker is a service and sales worker; 0 otherwise	Q5
Skilledagriculturalforestryfish	1 if the worker is a skilled agricultural, forestry and fish worker; 0 otherwise	Q5
Craftrades	1 if the worker is craft and related trades worker; 0 otherwise	Q5
Plantmachine	1 if the worker is a plant and machine operators, and assemblers; 0 otherwise	Q5
Elementaryocc	1 if the worker perform an elementary occupation; 0 otherwise	Q5

### The econometric models

The theoretical hypothesis regarding the association between working conditions and *SAH* is tested using a standard ordered probit model that is generally used to investigate discrete data of this kind. The model is built around a latent regression, which takes the following form:

$$y_{i}^{*}=x_{i}^{'}\beta +\varepsilon_{i}$$ (1)

where *x* and *β* are respectively the matrix of control variables and the vector of unknown parameters, *ε* is the error term, subscript *i* denotes an individual observation, and, as usual, y* is unobserved. We observe the following:

$$y=0\;\mathrm{if}\;y^{*}\leq 0$$ (2)

$$y=1\;\mathrm{if}\;0<y^{*}\leq \mu_{1}\ldots$$ (3)

$$y=J\;\mathrm{if}\;\mu_{J-1}\leq y^{*}$$ (4)

which is a form of censoring. Furthermore, *μ*′ is an unknown parameter to be estimated with *β*. We do not observe y* in the data. Rather, we observe the dependent variable, self-assessed health (*SAH*).

The theoretical hypothesis regarding the association between working conditions and *SICK*, which expresses the probability of having any illness or health problem that has lasted or is expected to last for more than 6 months is tested using a standard probit model that takes the following form:

$$P_{r}(Y_{i}=1)=\phi (x_{i}\beta )$$ (5)

where *ϕ* represents the cumulative normal distribution function, *x* is a vector of explanatory variables, *β* is a vector of parameter estimates, and subscript *i* denotes an individual observation.

As is known, the interpretation of the coefficients is quite difficult in the ordered probit, since neither their sign nor their magnitude is informative with respect to the partial effects of a given explanatory variable. Therefore, the interpretation of the coefficients is unclear [23]. For this reason, we calculate the marginal effects, which allow for interpreting the effect of the regressors on the dependent variable. Marginal effects measure the expected direct change in the dependent variable as a function of the change in a certain explanatory variable while keeping all other covariates constant. In an ordered probit model, marginal effects are difficult to interpret, since they are not equal to the coefficients, nor do their signs necessarily correspond to the signs of the coefficients [24]. However, the marginal effects of the regressors, which are expressed in terms of a change in the independent variables both on the probability of reporting good, fair and bad health and on the probability of being *SICK* provide an idea of the magnitude of the correlations between health and working conditions.

## Results and discussion

Table 2 reports the marginal effects (*dx/dy*) of a change in the regressors on the probability of reporting good, fair and bad health. For brevity, the results on country dummies are not reported in Tables 2 and 3 but only commented.

**Table 2:** The marginal effect (dx/dy) of a change in the regressors on the probability of reporting good, fair and bad health.

	*Good SAH*			*Fair SAH*			Bad SAH		
*Variable*	*dx/dy*	*SE*	*P > | z |*	*dx/dy*	*SE*	*P > | z |*	*dx/dy*	*SE*	*P > | z |*
*Demographic*									
Age	-.0072512***	.00026	0.000	.0065406 ***	.00024	0.000	.0007106***	.00004	0.000
Male	.0048208	.0065	0.458	-.0043486	.00586	0.458	-.0004722	.00064	0.458
Phd	-.0330277***	.00689	0.000	.0298484***	.00624	0.000	.0031793***	.00068	0.000
Npeople	.0057183**	.00228	0.012	-.0051579**	.00206	0.012	-.0005604**	.00023	0.013
Endmeet	.0513548***	.00619	0.000	-.0461977***	.00557	0.000	-.0051571***	.00069	0.000
*Job demand*									
Howmanyh	.0017444***	.0003	0.000	-.0015735***	.00027	0.000	-.0001709***	.00003	0.000
Notimef	-.0203197***	.00644	0.002	.018294***	.00579	0.002	.0020257 ***	.00066	0.002
Highspeed	-.0027798**	.00107	0.010	.0025074***	.00097	0.010	.0002724**	.00011	0.010
*Psychological environment*									
Stress1	-.0341282***	.01123	0.002	.0306239***	.01003	0.002	.0035043***	.00122	0.004
Stress2	-.0026281	.00907	0.772	.0023709	.00819	0.772	.0002572	.00089	0.772
Worrying	-.0378222***	.00636	0.000	.0340207***	.00572	0.000	.0038015***	.00068	0.000
Exhausted1	-.1229589***	.00966	0.000	.109152***	.00848	0.000	.0138069***	.00142	0.000
Exhausted2	-.0467442***	.00836	0.000	.0420403***	.00751	0.000	.0047039***	.00089	0.000
Satisfied	.0635434***	.00899	0.000	-.0565061***	.0079	0.000	-.0070373***	.00116	0.000
Inforisk	.0350474***	.00988	0.000	-.0313392 ***	.00876	0.000	-.0037082***	.00114	0.001
Hrisk	-.085717***	.00761	0.000	.0762722***	.0067	0.000	.0094447***	.00105	0.000
*Job hazard*									
Envirconds	.0013157***	.00039	0.001	-.0011868***	.00035	0.001	-.0001289***	.00004	0.001
Physconds	.0013672***	.0004	0.001	-.0012333***	.00036	0.001	-.000134***	.00004	0.001
*Job recognition*									
Manhelp1	.0265626**	.01202	0.027	-.0238959**	.01078	0.027	-.0026667**	.00125	0.033
Manhelp2	.0190144*	.0113	0.092	-.0171907*	.01024	0.093	-.0018237*	.00107	0.089
Adcareer1	.0303639***	.00718	0.000	-.0274456***	.00651	0.000	-.0029182***	.00069	0.000
Adcareer2	.0258458***	.00708	0.000	-.0234076***	.00644	0.000	-.0024382***	.00066	0.000
Recognition1	.029917***	.00851	0.000	-.0269218***	.00764	0.000	-.0029952***	.00089	0.001
Recognition2	.0091769	.00838	0.273	-.0082912	.00758	0.274	-.0008858	.0008	0.267
*Job characteristics*									
Private	-.0128752*	.00698	0.065	.0115918*	.00627	0.065	.0012834*	.00071	0.072
Other	-.0218282	.01322	0.099	.0195725	.01178	0.097	.0022557	.00144	0.118
Armedforces	.081835**	.03266	0.012	-.0755173**	.0308	0.014	-.0063177***	.0019	0.001
Managers	.0438689***	.01498	0.003	-.0400309***	.01383	0.004	-.003838***	.00117	0.001
Professionals	.0662943***	.01031	0.000	-.0605861***	.00956	0.000	-.0057082***	.00083	0.000
Technicians	.0380144***	.01076	0.000	-.0345733***	.00987	0.000	-.0034411***	.00091	0.000
Clerical	.0271571***	.00936	0.004	-.019332*	.01054	0.067	-.0019958*	.00104	0.054
Servicesales	.0082728	.02523	0.743	-.0245949***	.00851	0.004	-.0025622***	.00086	0.003
Skilledagriculturalforestryfish	.0082728	.02523	0.743	-.00748	.02287	0.744	-.0025622	.00086	0.737
Craftrades	.0270458***	.01016	0.008	-.024535***	.00927	0.008	-.0025108***	.0009	0.005
Plantmachine	.0225812**	.0112	0.044	-.020479**	.01021	0.045	-.0021022**	.00099	0.034

***stat. signf. at 1%

** stat. signf. at 5%

* stat. signf. at 10%.

**Table 3:** The marginal effect (*dx/dy*) of a change in the regressors on the probability of being *SICK*.

*Demographic*	*dx/dy*	*SE*	*P > | z |*
Age	.0048973***	.00024	0.000
Male	-.0222642***	.00614	0.000
Phd	.0043048	.00651	0.508
Npeople	-.004992**	.00213	0.019
Endmeet	-.019664***	.00597	0.001
*Job demand*			
Howmanyh	-.0018372***	.00027	0.000
Notimef	.0008029	.00607	0.895
Highspeed	.0019191*	.00102	0.059
*Psychological environment*			
Stress1	.0336422***	.01079	0.002
Stress2	.00644	.00856	0.452
Worrying	.0360862***	.0061	0.000
Exhausted1	.0532346***	.00863	0.000
Exhausted2	.013692*	.00744	0.066
Satisfied	-.0241742***	.00858	0.005
Inforisk	-.0136347	.00906	0.132
Hrisk	.0852611***	.00747	0.000
*Job hazard*			
Envirconds	-.001428***	.00038	0.000
Physconds	-.0016803***	.00037	0.000
*Job recognition*			
Manhelp1	-.0190184	.01138	0.095
Manhelp2	-.0313422***	.01052	0.003
Adcareer1	-.0295087***	.0066	0.000
Adcareer2	-.0289855***	.00673	0.000
Recognition1	-.0134817	.00819	0.100
Recognition2	-.0127863	.00834	0.125
*Job characteristics*			
Private	.0017264	.00642	0.788
Other	.0322634***	.01298	0.013
Armedforces	.0752722	.04795	0.116
Managers	-.0008318	.01599	0.959
Professionals	-.0085064	.01148	0.459
Technicians	.004657	.0115	0.685
Clerical	.008835	.01222	0.470
Servicesales	-.0057535	.00955	0.547
Skilledagriculturalforestryfish	-.0412724*	.02301	0.073
Craftrades	-.0223296**	.01033	0.031
Plantmachine	-.0170342	.01136	0.134

***stat. signf. at 1%

** stat. signf. at 5%

* stat. signf. at 10%.

As the number of hours that are usually worked per week rises, the probability of reporting good and fair health is expected to increase, while the probability of reporting bad self-assessed health is expected to decrease. Workers who found that, in the last 12 months, their job prevented them from giving the time they wanted to their family have a lower probability of reporting good health and a higher probability of reporting fair and bad health. As the working speed increases, the probability of reporting good health decreases while the probability of reporting fair and bad self-assessed health is expected to increase. Taking the workers who never experience stress in their work as the reference group, workers who experience stress always and most of the time have a lower probability of reporting good health and a higher probability of reporting fair and bad health. Workers who keep worrying about their work when they do not work have a lower probability of reporting good self-assessed health and a higher probability of reporting fair and bad self-assessed health. Workers who always, mostly and sometimes feel exhausted at the end of the day have a lower probability of reporting good health and a higher probability of reporting fair and bad self-assessed health with respect to workers who never feel exhausted. Workers who are satisfied with their working conditions have a higher probability of reporting good health and a lower probability of reporting fair and bad health. Being informed on the health and safety risks related to job performance is associated with a 3.5 percent higher probability of reporting good health and a 3.1 and 0.3 percent lower probability of reporting fair or bad health, respectively. Thinking that one’s own health or safety is at risk because of work is associated with an 8.5 percent lower probability of reporting good health, a 7.6 percent higher probability of reporting fair health and a 0.9 percent higher probability of reporting bad health.

As exposure to adverse environmental working conditions (both *Envirconds* and *Physconds*) decreases, the probability of reporting good health increases and the probability of reporting fair and bad health goes down. Workers who receive helps and support from their managers always/most of the time have a 2.6 percent higher probability of reporting good health and a 2.3 percent and 0.2 percent lower probability of reporting fair or bad health, respectively. Interviewees who think that their job offers good prospects for career advancement (*Adcareer1* and *Adcareer2*) have a higher probability of reporting good health and a lower probability of reporting fair or bad health. Workers who think that they receive the recognition they deserve at their job (*Recognition1*) have a 2.7 percent higher probability of reporting a good health and 2.6 or 0.2 percent lower probability of reporting fair or bad self-assessed health, respectively. Private sector workers have a lower probability of reporting good health and a higher probability of reporting fair or bad health, respectively, than do public workers. Taking elementary occupation workers as the reference group, armed forces workers, managers, professionals, technicians, clerical support workers, craft and related trades workers, and plant and machine operators and assemblers have a higher probability of reporting good health and a lower probability of reporting fair or bad health. With respect to country dummies, taking the UK as the reference group, workers from Austria, Belgium, Denmark, Estonia, Finland, Italy, the Netherlands, Poland, Romania, Slovakia, Slovenia, Sweden, and Portugal have a lower probability of reporting good health, while workers from Croatia, Cyprus, Greece, and Ireland have a higher probability of reporting good health.

Table 3 reports the marginal effect (*dx/dy*) of a change in the regressors on the probability of being *SICK* (having any illness or health problem that has lasted or is expected to last for more than 6 months).

As the number of hours that workers usually work per week rises, the probability of being sick is expected to decrease. As working speed increases, the probability of being sick is expected to increase. Taking the workers who never experience stress in their work as the reference group, workers who always and most of the time experience stress have a higher probability of being sick. Workers who keep worrying about their work when they are not working have a higher probability of being sick. Workers who always, mostly and sometimes feel exhausted at the end of the day have a higher probability of being sick. Workers that are satisfied with their working conditions have a lower probability of being sick. Thinking that one’s own health or safety is at risk because of work is associated with an 8.5 percent higher probability of being sick.

As exposure to adverse environmental working conditions (both *Envirconds* and *Physconds*) decreases, the probability of being sick decreases. Workers who sometimes and rarely receive help and support from their managers (*Manhelp2*) have a 3.1 percent lower probability of being sick than workers who never receive help and support from their manager. Interviewees who think that their job offers good prospects for career advancement (*Adcareer1*, *Adcareer2*) have a lower probability of being sick. Workers who think that they receive the recognition they deserve for their work (*Recognition1*) have a 1.3 percent higher probability of reporting good health. Interviewees working in joint private-public organizations or companies, the not-for-profit sector, NGOs and elsewhere have a higher probability of being sick than public workers. Taking elementary occupation workers as the reference group, skilled agricultural, forestry, fishing, craft and related trades workers have a lower probability of being sick. With respect to the country dummies, taking the UK as the reference group, workers from Belgium, Denmark, Estonia, Finland, France, Latvia, Luxemburg, the Netherlands, Slovenia, and Sweden have a higher probability of being sick, while workers from Croatia, Cyprus, Greece, Hungary, Ireland, Italy, Poland, Romania, Slovakia, Portugal and Spain have a lower probability of being sick.

## Discussion

The major limitation of the econometric analyses is that the findings define a correlation rather than a cause-and-effect relation between health (as measured by *SAH* and *SICK*) and working conditions, and association does not equal causation. The analyses that are implemented do not allow for us to ascertain a clear causal relationship in one direction or the other. Therefore, causation could go in both directions, with the healthier workers having more opportunities for good jobs that are characterized by favourable working conditions, and the good working conditions improve workers’ health.

The results indicate that, in the EU28, there is a positive association between (both subjective self-assessed and more objective) health and good working conditions. Although there are limitations to the empirical models that are estimated (without instrumental variables) and the data that are employed (cross sectional data), the results allow for making a comparison between the two different measures of health and they seem to support the three main dimensions (demand, control and rewards) of the Demand-Control-Support model [6] and the Effort-Reward Imbalance model [7].

The results for the socio demographic variables are in line with the main literature on health [25–18]. 1) Being older decreases the probability of reporting good *SAH* and increases the probability of being *SICK*. 2) Males have a lower probability of being *SICK*. 3) Having a doctoral degree and/or a Phd decreases the probability of reporting good *SAH* and increases the probability of being *SICK*, meaning that higher educated people that have higher expectations on their health are likely to see these expectations unmet with undesirable effects on health. This result is not in line with the literature (see, among others, [11– 14]). 4) More people living in the household increases the probability of reporting good *SAH* and decreases the probability of being *SICK*. This result is likely because the larger that a family, the more copious the psychological recourses that are available to family members that have positive effects on health. 5) Interviewees whose household total monthly income is able to make ends meet have a higher probability of reporting good *SAH* and a lower probability of being *SICK*. With respect to job demands, more hours worked improves *SAH* and decreases the probability of being *SICK*. This result is not in line with the literature (see, among others, [14]). However, it could be explained that more working hours implies decreasing leisure time, which is a way to improve well-being [26], and increases income, which is likely to be instrumental to a better life style and, therefore, to better health. The literature reports that when people work more hours than they want, it is likely to have adverse health consequences, but the absolute number of hours worked does not seem to be harmful to health [27]. As the working speed increases, the probability of reporting a good *SAH* decreases [18], and the probability of being *SICK* is expected to increase.

With respect to proxies for the psychological environment, when they are significant for both measures of health, the results are similar with no difference between reported health and objective health. The result for satisfaction with working conditions is interesting and in line with the literature [13–14], since increasing satisfaction with working conditions improves *SHA* and reduces the probability of being *SICK*. This is likely to happen, since work satisfaction plays an important role in determining the overall quality of life [28]. With respect to the working environment, decreasing exposure to adverse environmental conditions improves *SHA* [18] and reduces the probability of being *SICK*. As expected, and as identified by Siegrist [7], more recognition is good for workers’ well-being and is correlated with better self-rated health. Receiving help and support from their managers [10–15] and thinking that one’s own job offers good prospects for career advancement [7–9–18] is likely to be good for both subjective and objective health. Job differences are significant for health, which is in line with the literature [18], since higher positions are associated with a higher probability of reporting good health than elementary occupations. With respect to sectors, as in previous studies [18], the results show that private sector workers have a lower probability of reporting good health.

## Conclusion

The study investigates the correlation between working conditions and two measures of health, *SAH* (a subjective measure) and *SICK* (selected as a more objective measure), by employing data from the Sixth European Working Conditions Survey (2017). The definition of working conditions that is adopted in the paper is a broad one; however, the “Demand-Control-Support” model [6] and the “Effort-Reward-Imbalance” model [7] have been considered as theoretical references.

The results show that a mostly encouraging work environment, as well as good working conditions and job support, are associated with both better reported health and objective health [6– 7– 11–12]. The consequences of poor working conditions are pricy for individuals, since they may result in dangerous health effects, lead to absenteeism [29] and retirement, increase pension costs, and decrease worker productivity. In addition, poor working conditions can also cause occupational accidents that are costly for society; moreover, sickness, absenteeism and anticipated retirements imply increasing welfare costs [30]. Therefore, working conditions should be constantly and accurately monitored, and governments should not underestimate the overall (individual and collective) profitability of investments in their improvements. If improving working conditions is costly, the consequences of poor working conditions could be even more costly than improving working conditions themselves. Indeed, it seems that the calculations of the costs related to their improvements are easier than the calculations of the costs associated with the overall consequences of poor working conditions.

## Supporting information

S1 File Table A. Descriptive statistics explanatory variables. Table B. Descriptive statistics self-assessed health. Table C. Descriptive statistics self-assessed health (*SAH*). Table D. Descriptive statistics the occurrence of any illness or health problem, which has lasted, or is expected to last, for more than 6 months (*SICK*).
(DOCX)
