# Supplementary material for: Is there an association between working conditions and health? An analysis of the Sixth European Working Conditions Survey data
Source: PLoS One. 2019 Feb 12;14(2):e0211294. doi: 10.1371/journal.pone.0211294 (PMC6372138; doi:10.1371/journal.pone.0211294)
Supplement: S1 File — Table A. Descriptive statistics explanatory variables. Table B. Descriptive statistics self-assessed health. Table C. Descriptive statistics self-assessed health (SAH). Table D. Descriptive statistics the occurrence of any illness or health problem, which has lasted, or is expected to last, for more than 6 months (SICK). (DOCX) [file pone.0211294.s001.docx]

**Supporting Information**

**Table A. Descriptive statistics explanatory variables.**

| ***Variable*** | Mean | Std. Dev. | Min | Max |
| --- | --- | --- | --- | --- |
|  |  |  |  |  |
| *Demographic* |  |  |  |  |
| Age | 42.91525 | 12.23026 | 15 | 89 |
| Male | .4784071 | .4995439 | 0 | 1 |
| Phd | .6215667 | .4850074 | 0 | 1 |
| Npeople | 2.787157 | 1.300328 | 0 | 10 |
| Endmeet | .5626492 | .4960699 | 0 | 1 |
| *Job demand* |  |  |  |  |
| Howmanyh | 36.62935 | 11.10406 | 1 | 105 |
| Notimef | .3433464 | .4748359 | 0 | 1 |
| Highspeed | 3.291955 | 2.915309 | 0 | 7 |
| *Workers sensations* |  |  |  |  |
| Stress1 | .2561103 | .4364926 | 0 | 1 |
| Stress2 | .590892 | .4916795 | 0 | 1 |
| Stress3 | .1529977 | .3599928 | 0 | 1 |
| Worrying | .3706767 | .4829961 | 0 | 1 |
| Exhausted1 | .3361621 | .4724049 | 0 | 1 |
| Exhausted2 | .4187778 | .4933691 | 0 | 1 |
| Exhausted3 | .2450601 | .4301318 | 0 | 1 |
| Satisfied | .8462886 | .3606794 | 0 | 1 |
| Inforisk | .8964239 | .3047163 | 0 | 1 |
| Hrisk | .2632246 | .4403926 | 0 | 1 |
| *Job hazard* |  |  |  |  |
| Envirconds | 55.96516 | 8.371939 | 1 | 7 |
| Physconds | 45.81127 | 8.254937 | 1 | 7 |
| *Job recognition* |  |  |  |  |
| Manhelp1 | .633146 | .4819565 | 0 | 1 |
| Manhelp2 | .2992835 | .457954 | 0 | 1 |
| Manhelp3 | .0675705 | .2510128 | 0 | 1 |
| Adcareer1 | .3555382 | .4786865 | 0 | 1 |
| Adcareer2 | 2281754 | .4196654 | 0 | 1 |
| Adcareer3 | .4162864 | .4929529 | 0 | 1 |
| Recognition1 | .6153717 | .4865175 | 0 | 1 |
| Recognition2 | .2022944 | .401719 | 0 | 1 |
| Recognition3 | .1823339 | .3861276 | 0 | 1 |
| *Job characteristics* |  |  |  |  |
| Private | .2470146 | .4312843 | 0 | 1 |
| Public | .6936534 | .460985 | 0 | 1 |
| Other | .0593319 | .2362499 | 0 | 1 |
| Armedforces | .0047445 | .0687179 | 0 | 1 |
| Managers | .0376228 | .1902861 | 0 | 1 |
| Professionals | .1298069 | .3360978 | 0 | 1 |
| Technicians | .1158232 | .3200195 | 0 | 1 |
| Clerical | .1060846 | .3079522 | 0 | 1 |
| Servicesales | .2437989 | .4293817 | 0 | 1 |
| Skilledagriculturalforestryfish | .0118195 | .1080756 | 0 | 1 |
| Craftrades | .1311387 | .337559 | 0 | 1 |
| Plantmachine | .0889795 | .2847201 | 0 | 1 |
| Elementaryocc | .1301815 | .33651 | 0 | 1 |
| *Countries* |  |  |  |  |
| Austria | .0305534 | .1721078 | 0 | 1 |
| Belgium | .0727718 | .259767 | 0 | 1 |
| Bulgaria | .0363236 | .187098 | 0 | 1 |
| Croatia | .0339159 | .1810165 | 0 | 1 |
| Cyprus | .0337498 | .1805883 | 0 | 1 |
| Czech Republic | .02607 | .1593468 | 0 | 1 |
| Denmark | .0305534 | .1721078 | 0 | 1 |
| Estonia | .0322554 | .1766813 | 0 | 1 |
| Finland | .0324214 | .1771203 | 0 | 1 |
| France | .037569 | .1901554 | 0 | 1 |
| Germany | .0760928 | .2651521 | 0 | 1 |
| Greece | .0263606 | .1602085 | 0 | 1 |
| Hungary | .0242019 | .1536788 | 0 | 1 |
| Ireland | .0286438 | .1668067 | 0 | 1 |
| Italy | .0367388 | .1881236 | 0 | 1 |
| Latvia | .0347461 | .18314 | 0 | 1 |
| Lithuania | .0200506 | .1401764 | 0 | 1 |
| Luxembourg | .0171863 | .1299677 | 0 | 1 |
| Malta | .0365312 | .1876116 | 0 | 1 |
| Netherlands | .0196355 | .1387471 | 0 | 1 |
| Poland | .0326705 | .1777764 | 0 | 1 |
| Portugal | .0300552 | .1707428 | 0 | 1 |
| Romania | .0234132 | .1512149 | 0 | 1 |
| Slovakia | .0268587 | .1616738 | 0 | 1 |
| Slovenia | .0550044 | .2279935 | 0 | 1 |
| Spain | .0812404 | .2732096 | 0 | 1 |
| Sweden | .0321724 | .1764613 | 0 | 1 |
| UK | .0322139 | .1765713 | 0 | 1 |

**Table B. Descriptive statistics self-assessed health.**

|  | Freq. | Percent | Cum. |
| --- | --- | --- | --- |
| Very good | 6,040 | 25.10 | 25.10 |
| Good | 12,479 | 51.86 | 76.96 |
| Fair | 4,902 | 20.37 | 97.34 |
| Bad | 574 | 2.39 | 99.72 |
| Very bad | 67 | 0.28 | 100.00 |
| Total | 24,062 | 100.00 |  |

**Table C. Descriptive statistics self-assessed health (*SAH*).**

| *SAH* | Freq. | Percent | Cum. |
| --- | --- | --- | --- |
| Very good and good | 18,519 | 76.96 | 76.96 |
| Fair | 4,902 | 20.37 | 97.34 |
| Bad and very bad | 641 | 2.66 | 100.00 |
| Total | 24,062 | 100.00 |  |

**Table D. Descriptive statistics the occurrence of any illness**

**or health problem, which has lasted, or is expected to last,**

**for more than 6 months (*SICK*).**

| *SICK* | Freq. | Percent | Cum. |
| --- | --- | --- | --- |
| Yes | 4,369 | 18.24 | 18.24 |
| No | 19,589 | 81.76 | 100.00 |
| Total |  | 100.00 |  |
